# Neutron Imaging Using a Fine-Grained Nuclear Emulsion

**DOI:** 10.3390/jimaging7010004

**Published:** 2021-01-05

**Authors:** Katsuya Hirota, Tomoko Ariga, Masahiro Hino, Go Ichikawa, Shinsuke Kawasaki, Masaaki Kitaguchi, Kenji Mishima, Naoto Muto, Naotaka Naganawa, Hirohiko M. Shimizu

**Affiliations:** 1 Department of Physics, Nagoya University, Furo-cho, Chikusa, Nagoya 464-8602, Japan; kitaguchi@phi.phys.nagoya-u.ac.jp (M.K.); muto@flab.phys.nagoya-u.ac.jp (N.M.); naganawa@flab.phys.nagoya-u.ac.jp (N.N.); shimizu@phi.phys.nagoya-u.ac.jp (H.M.S.); 2 Faculty of Arts and Science, Kyushu University, Motooka Nishi, Fukuoka 819-0395, Japan; tomoko.ariga@cern.ch; 3 Institute for Integrated Radiation and Nuclear Science, Kyoto University, Kumatori, Osaka 590-0494, Japan; hino.masahiro.2x@kyoto-u.ac.jp; 4 High Energy Accelerator Research Organization, Tokai, Ibaraki 319-1106, Japan; go.ichikawa@kek.jp (G.I.); shinsuke.kawasaki@kek.jp (S.K.); kenji.mishima@kek.jp (K.M.); 5 Kobayashi-Maskawa Institute for Origin of Particles and the Universe (KMI), Nagoya University, Furo-Cho, Chikusa, Nagoya 464-8602, Japan; 6 Institute of Materials and Systems for Sustainability, Nagoya University, Furo-Cho, Chikusa, Nagoya 464-8602, Japan

**Keywords:** neutron imaging, nuclear emulsion, cold neutron

## Abstract

A neutron detector using a fine-grained nuclear emulsion has a sub-micron spatial resolution and thus has potential to be applied as high-resolution neutron imaging. In this paper, we present two approaches to applying the emulsion detectors for neutron imaging. One is using a track analysis to derive the reaction points for high resolution. From an image obtained with a 9 μm pitch Gd grating with cold neutrons, periodic peak with a standard deviation of 1.3 μm was observed. The other is an approach without a track analysis for high-density irradiation. An internal structure of a crystal oscillator chip, with a scale of approximately 30 μm, was able to be observed after an image analysis.

## 1. Introduction

Photographic emulsions made of silver halide crystals dispersed in gelatin were used in film batches and has been used in X-ray imaging because ionization by charged particles can be visualized as shading of silver grains through the development process (photographic processing). A neutron detector that combines an X-ray film and a converter was developed for neutron imaging and has since been widely used in neutron facilities for recording image of structures as fine as 100 μm in a large area. Later, with the development of digital imaging devices such as charge-coupled devices (CCDs), photographic emulsion detectors are not being used in neutron imaging applications because of the difficulties associated with real-time measurements. In recent years, energy-selective neutron transmission imaging with detectors that can measure neutron times of flight (TOFs) has become extremely popular [1,2].

In the field of nuclear particle physics, photographic emulsions continued to be developed; the size, sensitivity, and ratio of silver halide crystals in gelatin were tuned for recording the charged particle tracks. These emulsions, called nuclear emulsions, have track-recording capabilities and sub-micrometer resolutions suitable for nuclear and high-energy particle physics experiments such as hyper-nuclear experiments [3,4] and tau-neutrino detection [5,6].

Because the typical spatial resolution of a nuclear emulsion is related to the average distance between grains, a spatial resolution of less than 100 nm can be achieved using a fine-grained nuclear emulsion. A Silver halide with a grain size of 40 nm is used for preparing a fine-grained nuclear emulsion to search for dark matter [7,8,9]. The sensitivity of these emulsions is adjusted such that they are not sensitive to minimum ionizing particles and gamma rays. We have studied the use of these emulsions as neutron detectors for fundamental physics experiments [10,11]. We used boron as a neutron converter and irradiated it with cold and ultra- cold neutrons, achieving a spatial resolution of up to 11 nm. This detector is not suitable for applications in which neutron energy measurement is required, but it is expected to be useful in applications such as neutron imaging. In this study, transmitted images of industrial products were measured and analyzed for future applications.

The neutron reaction point must be determined by track analysis to achieve a high spatial resolution. The calculated ranges in the emulsion layer were 4.9 μm for α particles and 2.5 μm for Li. These values were calculated using SRIM based on the composition of the emulsion described in [8], assuming a uniform dispersion of silver halide crystals in gelatin owing to its protective colloidal properties, and averaging over the composition throughout the volume of the emulsion layer. Because the track is approximately 5 μm, it is possible to use this detector for image detection with a spatial resolution of less than 5 μm without track analysis. In this case, the image is developed(photoprocessed) and then acquired with an epi-illumination optical microscope. The highest spatial resolution of current neutron imaging is approximately 5 μm [12,13,14]. The spatial resolution of the emulsion detector is potentially comparable to that of existing neutron detectors without track analysis. If high-resolution images are required, the resolution can be further improved by track analysis. The detector has potential applications in transmission image for ultra-precise measurements.

Because the nuclear emulsion itself is not sensitive to neutral particles, the neutrons must be converted into charged particles. The candidate materials for the converter are ${}^{10}$B and ${}^{6}$Li. Gadolinium, which has a large neutron absorption cross section, cannot be used in the converter because the detector is not sensitive to gamma rays. The detection efficiency of ${}^{10}$B is higher than that of ${}^{6}$Li because its neutron absorption cross section is larger. The range of t-particles from Li is approximately 40 μm, and it is easy for the tracks to overlap each other. Moreover, owing to the low grain density of t-particles, the signal-to-noise ratio is worse than the α and ${}^{7}$Li traces by ${}^{10}$B absorption. From these perspectives, using ${}^{10}$B as the a neutron converter is more advantageous for our detector. In this study, a B${}_{4}$C layer is deposited with a thickness of up to 2 μm. The reasons for this are as follows: If a thicker B${}_{4}$C layer is used, the Li particles emitted after reaction with the neutrons will be stopped inside the B${}_{4}$C layer, the positional accuracy of the reaction point will be reduced when the track angle is nearly parallel to the B${}_{4}$C layer, and the B${}_{4}$C layer may be unstable under the current sputtering method if it is too thick. To detect neutrons efficiently with a thin B${}_{4}$C layer, longer-wavelength neutrons should be used. In addition, it is easier to strengthen the contrast in high-spatial-resolution measurements when using longer-wavelength neutrons, as it is often necessary to observe subtle changes in the internal structure of a sample. Considering these facts, we developed a cold neutron imaging system.

**Figure 1:**
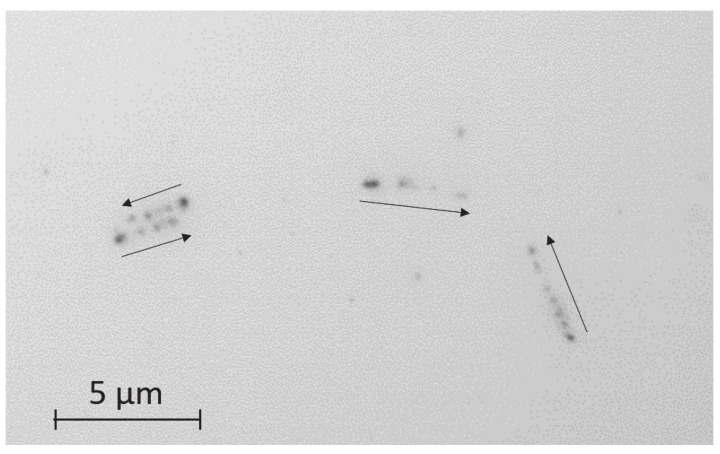
Typical track image of the nuclear emulsion. The arrows show the tracking direction.

Figure 1 shows a typical image of an emulsion detector irradiated by neutrons. The thickness of the B${}_{4}$C layer is 50 nm [11] in this case. We can see the tracks consisting of silver grains in this image. In each track, the emitted particles are moving in the direction shown by the arrow. Here, an alpha particle with a range of 4.9 μm and a Li particle with a range of 2.5 μm can be seen. In the nuclear emulsion detector, the energy loss corresponds to the linear density of silver grains. The grain size near the starting point of the track appears large owing to the microscope focus. Because the emitted particles spread isotropically from the reaction point, the two-dimensional image of the photograph does not directly correspond to the particle range. By setting a shallow depth of field and changing the focal point in the depth direction, we can obtain three-dimensional tracking information. We know that the neutron reaction point is located in the B${}_{4}$C layer, so we can focus on the volume near the layer for imaging and observe around the reaction points intensively.

There are similar types of track detectors, based on nitrocellulose films in combination with BN as a converter and a CR39 detector. These etching-type detectors have difficulties in improving the final spatial resolution because etching makes their tracks thicker. The tracks in our detector are made of silver grains with diameters of approximately 100 nm, so it is easy to evaluate the tracks accurately from the center of the grain.

Although the detector has a small mass, is thin, and does not use an electrical supply line, it has no real-time capability and is a low-throughput detector; therefore its effectiveness during use should be considered. One possible way to use this detector is to combine it with an existing scintillator and camera system, and another way is to consider using it in places other than imaging beamlines. This thin emulsion detector can also be placed directly upstream of the scintillator plate. The scintillator and camera system ensures real-time performance, and the emulsion detector functions as an optional detector to achieve high spatial resolution. Further, detection with a high spatial resolution does not always require a large-area beamline. High-intensity and low-divergence beams are needed. It may be possible to install an emulsion detector downstream of regular scattering measurements and conduct long-time irradiation. This thin, light, and size-adjustable emulsion detector can easily be set up anywhere.

## 2. Experimental Conditions

**Figure 2:**
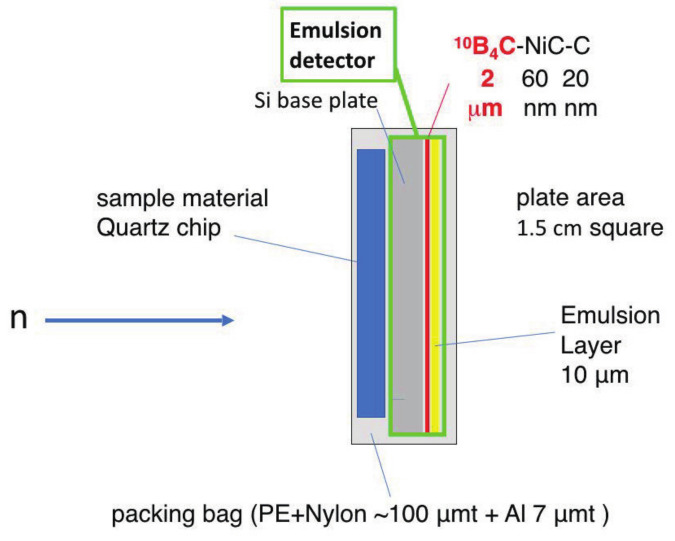
Structure of the neutron emulsion. The emulsion and sample material are vacuum-packed in a bag consisting of polyethylene, nylon, and aluminum.

Details of the neutron emulsion detector are described in [11]. After depositing a 2 μm thick ${}^{10}$B${}_{4}$C layer on a Si substrate of thickness 400 μm and coating it with NiC and C layers, an emulsion layer with a thickness of approximately 10 μm is obtained (see Figure 2). The B${}_{4}$C, NiC, and C layers were deposited using an ion sputtering system [15]. NiC is deposited for stabilizing the B${}_{4}$C layer and C is for better adhesion of the nuclear emulsion gel to the sputtered layer. To achieve high detection efficiency in imaging measurements, the thickness of the B${}_{4}$C is changed from that in the previous study [11]. The sample material is placed in close contact with the Si-substrate side to prevent damage to the emulsion layer. The cold neutrons incident from the Si-substrate side are absorbed by the ${}^{10}$B${}_{4}$C layer. Because the kinetic energy of the incident neutron is sufficiently small compared to the Q value of the reaction, the α and Li particles are emitted in opposite directions.

One of the two emitted particles enters the emulsion layer, whereas the other is absorbed by the Si substrate. The charged particles form a latent image in silver halide crystals in the emulsion layer when they enter it. The latent image can be confirmed as silver aggregates by developing this emulsion, and the size of each aggregate is approximately 100 nm. The alignment of the silver aggregates can be recognized as tracks.

The measurement is performed at a low-divergence beam branch of BL05 in the Materials and Life Science Experimental Facility (MLF) of Japan Proton Accelerator Research Complex (J-PARC) [16,17]. This BL05 uses a coupled moderator beamline. The beam branch provides neutrons with wavelengths of 0.2–1 nm. The sample materials are a 9 μm pitch Gd grating [18] (Slit No. 3) and a commercial crystal oscillator chip manufactured by KYOCERA Corporation. The Gd grating has a 9 μm pitch period and a thickness of 12 μm, and the opening space is 3 μm. The Gd grating is used to evaluate the spatial resolution of the imaging measurements, and the crystal chip is chosen to provide a practical example of future industrial product imaging. In this BL05 beam branch, the beam divergence can be adjusted using an upstream collimator. In the case of the Gd grating, the irradiation is measured at the divergence of 0.11 mrad to confirm the grating pitch, and in the case of the crystal-chip irradiation, the maximum possible divergence to enhance the neutron beam intensity, i.e., 10 mrad vertical and 1.4 mrad horizontal, is used. The neutron beam spread from the divergence is estimated as 0.1 μm because the distance between the Gd grating and the B${}_{4}$C layer is 900 μm. This beam spread is sufficiently smaller than the grating pitch. The beam power of the MLF is 533 kW, and the beam flux at that time is expected to be $7.6\times {\mathrm{10}}^{4}$ cm^−2^s^−1^ for the Gd grating measurement and $5.3\times {\mathrm{10}}^{5}$cm^−2^s^−1^ for the crystal-chip measurement. Under these conditions, both samples are irradiated for 3 h. The detection efficiency of the emulsion is estimated to be 5% from the B${}_{4}$C thickness, and it is estimated that $6\times 10^{7}$ tracks for the Gd grating measurement and $3\times 10^{8}$ tracks for the crystal-chip measurement are created in an area of 1 cm^2^. The expected densities of the silver particles are $6\times 10^{8}$ cm^−2^ and $3\times 10^{9}$ cm^−2^ assuming 10 are precipitated per trajectory [11].

## 3. Image Processing

**Figure 3:**
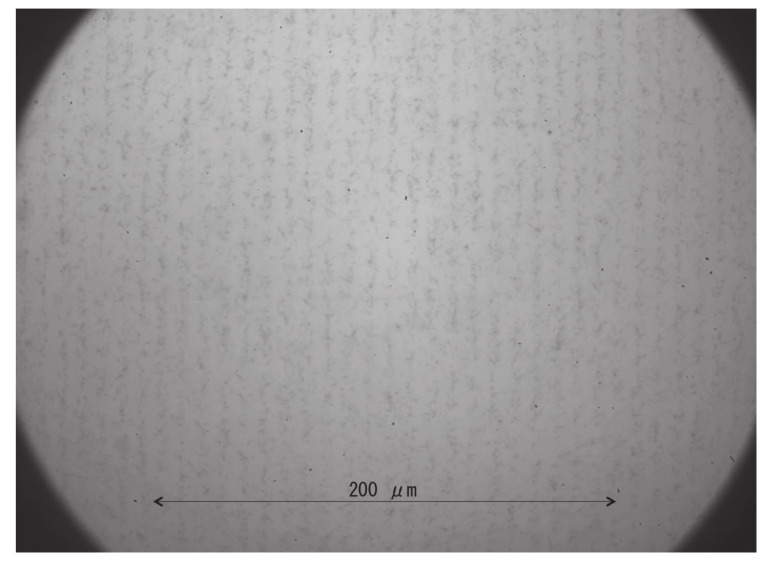
Neutron transmission image of the 9 μm pitch Gd grating. The black vertical stripes show the track marks on emulsion. There are 22 vertical stripes in this 200 μm area. These lines correspond to the grating pitch of 9 μm.

**Figure 4:**
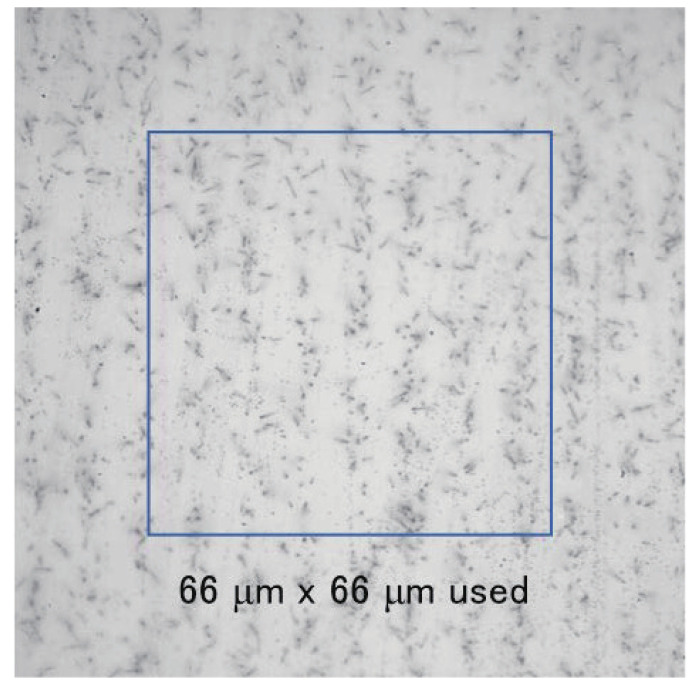
Expansion of the neutron transmission image of the Gd grating.

**Figure 5:**
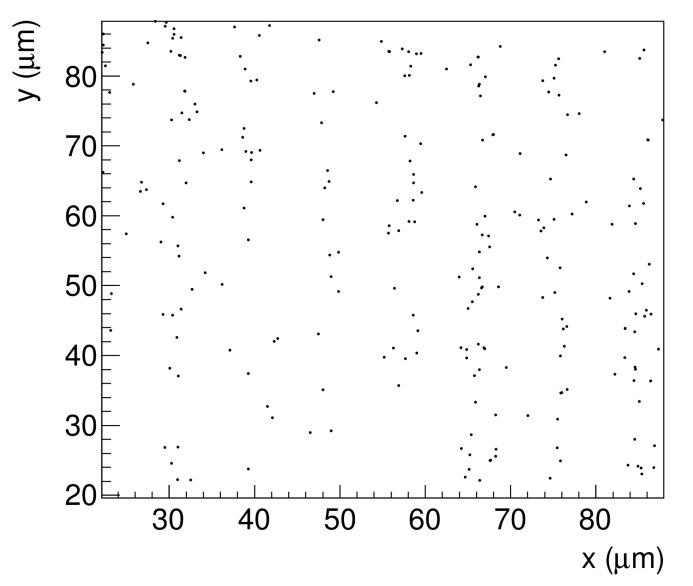
Starting points of tracks on the surface of the substance.

After neutron irradiation, an emulsion detector was developed (photoprocessed) at Nagoya University. Subsequently, the emulsion film was imaged using an epi-illumination optical microscope. The measured image of the 9 μm pitch Gd grating is shown in Figure 3. In this image, the track area is alternately seen with and without tracks, and the period is approximately 9 μm. An enlarged image was also captured using an epi-illumination optical microscope with a CMOS camera (see Figure 4). The pixel pitch was 54 nm. In this image, individual tracks can be recognized, but the tracks are close to each other and many of them intersect. We performed track analysis [11] for a 66×66$\mu m^{2}$ region of this image, with the start of the tracks shown in Figure 5. Because the track is not visible in the boron layer, the neutron reaction point and the starting point of each track is slightly different.

To use this emulsion detector as an imaging device, we need a statistics to discuss the transmission of the material, for which this image is insufficient. We performed a Rayleigh test [19,20] to check the periodicity of Figure 5. Here, we obtained the periodicity *d* and rotation α for each of the *n* tracks at the maximum of the following equations F(d,α) for the starting point coordinates $\left(x_{i},y_{i}\right)$:

(1) $$\begin{array}{c} F\left(d,\alpha \right)=\frac{2}{n}\left({\left(\sum_{i}^{n}\sin\left(\frac{2\pi }{d}\;\cdot \;x_{i}^{\prime }\right)\right)}^{2}+{\left(\sum_{i}^{n}\cos\left(\frac{2\pi }{d}\;\cdot \;x_{i}^{\prime }\right)\right)}^{2}\right), \end{array}$$

where

(2) $$\begin{array}{c} x_{i}^{\prime }=x_{i}\cos\alpha +y_{i}\sin\alpha . \end{array}$$

**Figure 6:**
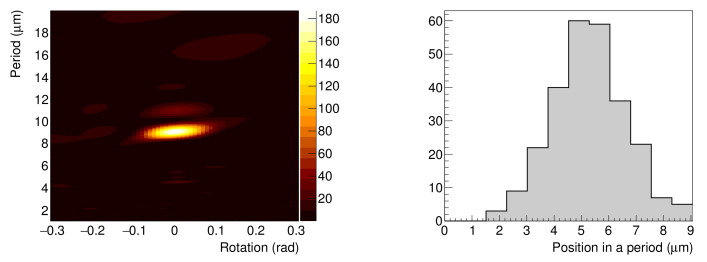
(**Left**) Results of the Rayleigh test. (**Right**) Distribution of the starting points of the tracks folded with the period of 9.06 μm.

Figure 6 (left) shows the F(d,α) value distribution. When d=9.06± 0.06 μm, the value of F(d,α) is the maximum. We can confirm that this figure reproduces the periodicity of the grating. The histogram of the distribution of the starting point of the tracks folded with the period of 9.06 μm is given in Figure 6 (right), with a standard deviation of 1.3 μm. The corresponding full width half maximum (FWHM) value is 3.1 μm, which is almost same as the aperture width of 3 μm, indicating that achievable resolution in neutron imaging is better than 3 μm.

**Figure 7:**
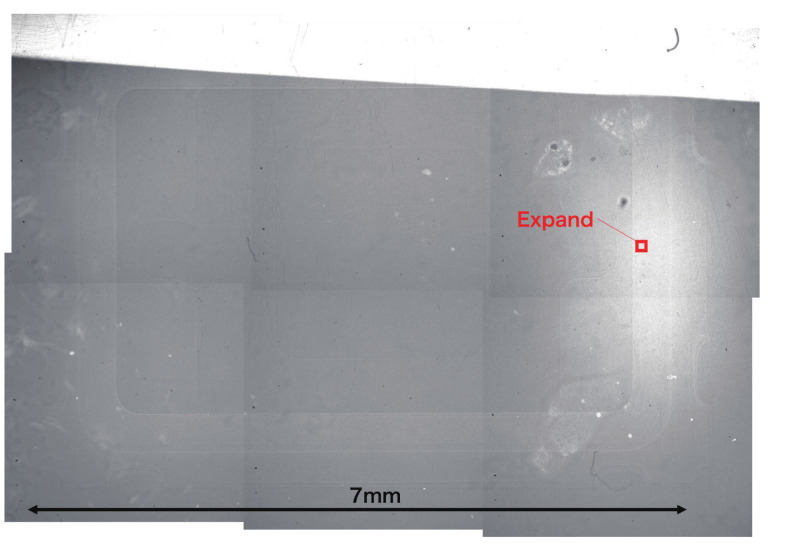
Transmission image of the quartz crystal oscillator taken using the emulsion detector. The area enclosed by the red square is enlarged and shown in Figure 8.

**Figure 8:**
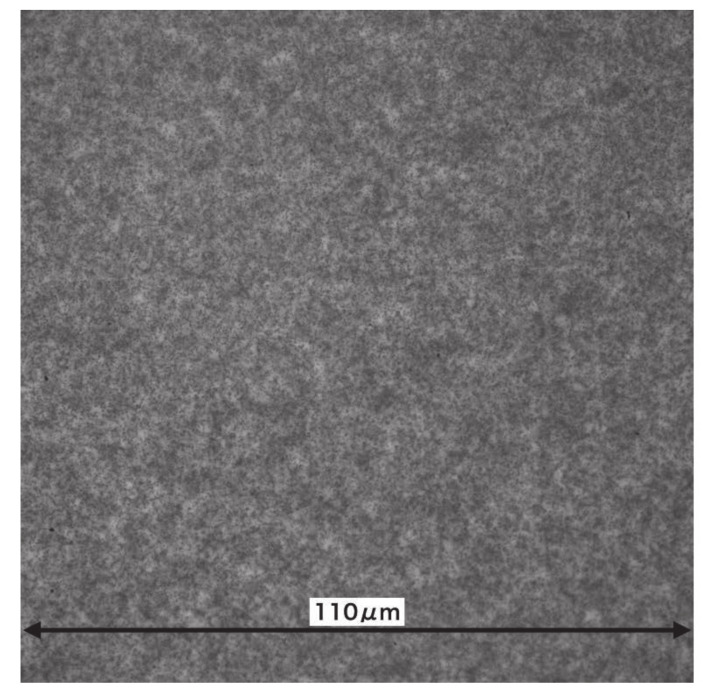
Expanded view of Figure 7. The silver grains are visible as black dots.

The image of the crystal chip is shown in Figure 7. It was taken at low magnification. The pixel pitch was 1.4 μm. A single field of view was approximately 2.8 mm at most; therefore, it was not possible for the whole image to be captured in one field of view when measuring the crystal chip. Six images were pasted together with an error of approximately 0.7 μm after adjusting the brightness in Figure 7. The area in which the neutrons were detected is blackened by tracks, which indicates that the area with low neutron transmission appears white, and that with high neutron transmission appears black. The right center of the image is white, and the number of silver grains is reduced in this area. This may be because the emulsion in this area is thicker than in the others, resulting in weaker development and suppressed silver grain growth. A method must therefore be devised to ensure that the emulsion is applied uniformly. Figure 8 is an enlarged image of the right-hand side of the red square area in Figure 7. Although the silver grains are visible, it is difficult to identify the tracks because the grains exist very densely, even for the area with the suppressed growth of silver grains. It is difficult to identify the reaction points from the individual track information using this analysis. On the other hand, it is possible to discriminate the tracks’ shading by the difference in neutron transmittance, and it is possible to obtain images with a spatial resolution of the range.

**Figure 9:**
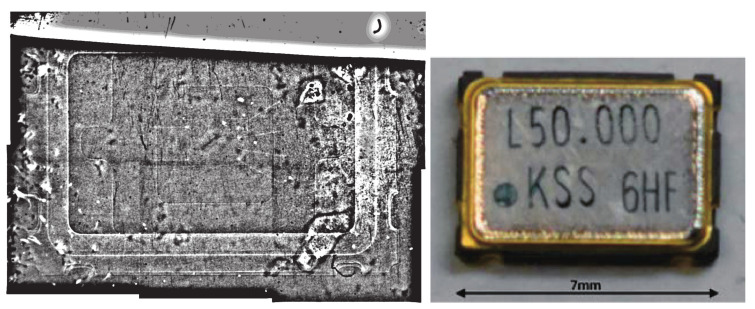
(**left**) Bandpass filter applied to the quartz crystal oscillator image. The vertical axis of the neutron beam corresponds to the horizontal axis of the oscillator image. (**right**) Photographic image of the quartz crystal oscillator.

In Figure 7, the periphery of the crystal chip can be seen, but its inner structure cannot be seen. Therefore, we used an image-processing technique to view the internal structure of the crystal chip, as a next step.The image has position-dependent components such as the beam intensity and detection efficiency. In the scintillator and camera system, these position dependencies can be cancelled out by dividing the image with the direct beam image. However, this is not possible in this detector. Therefore, the contrast was improved by removing the position-dependent components in another way.Because such positional dependencies were considered as fluctuations over a longer distance than the structure of the measured sample, we sharpened the image by reducing the periodic component over a longer distance using a bandpass filter. We used the fast Fourier transform (FFT) function of ImageJ [21] as a bandpass filter, with the result shown in Figure 9. The frequency corresponding to that from 3 to 40 pixels was transmitted in the FFT. In this image, four Au bonding wires can be seen from the center to the right side. The widths of these wires are estimated to be approximately 30 μm. The divergence angle of the beam used in this measurement is approximately 10 mrad. Considering that the thickness of the crystal chip is approximately 2 mm, a resolution of 30 μm wire is reasonable. In the future, a beam with a smaller divergence angle will be desirable to obtain a more detailed image.

## 4. Conclusions

A neutron emulsion detector has been developed and used to perform transmission image measurement for future industrial applications. A Gd grating and quartz crystal chips were used as the measurement samples. In the Gd grating measurement, tracks were analyzed. The grating spacing of the 9 μm pitch was confirmed using the Rayleigh test, and the standard deviation was 1.3 μm. The corresponding full width half maximum (FWHM) value is 3.1 μm, which is almost same as the aperture width of 3 μm, indicating that achievable resolution in neutron imaging is better than 3 μm.

For the quartz crystal-chip, irradiation was done by higher density to improve the statistics of neutron imaging. By using a bandpass filter, it was possible to observe the internal structure of the quartz crystal-chip. This result is expected to facilitate the acquisition of images with high spatial resolution. The quality of coating and drying of the emulsion layer needs to be improved for actual use. When the neutron irradiation is increased to improve the statistics of neutron imaging, the tracks overlap each other. Many overlapping tracks make it difficult to distinguish reaction points using the current track analysis method. By developing algorithms for track analysis at a high track density, images can be acquired with sub-micrometer spatial resolution.

## Data Availability

The data is presented within the article.
